# In-vivo study of osseointegration in Prestige LP cervical disc prosthesis

**DOI:** 10.1186/s12891-018-1957-2

**Published:** 2018-02-07

**Authors:** Jigang Lou, Beiyu Wang, Tingkui Wu, Wenjie Wu, Huibo Li, Ziyang Liu, Hao Liu

**Affiliations:** 1 Department of Orthopedics, West China Hospital, Sichuan University, 37 Guoxue Road, Chengdu, Sichuan 610041 China; 2 Department of Orthopedics, Southwest Hospital, the Third Military Medical University, Chongqing, China; 3 Department of Orthopedics, Qianfoshan Hospital, Shandong University, Jinan, Shandong China

**Keywords:** Cervical disc arthroplasty, Animal model, Porous ingrowth, Osseointegration, Histomorphometry

## Abstract

**Background:**

A study was designed to quantify the extent of porous osseointegration at the prosthesis-bone interface in the Prestige LP prosthesis containing a plasma-sprayed titanium coating.

**Methods:**

Using an anterior surgical approach, cervical disc arthroplasty was performed in 8 mature male goats at the C3-C4 segment, followed by implantation of the Prestige LP prosthesis. The vertebral specimens were examined using microcomputed tomograph for histomorphometric quantification, and proceeded by routine paraffin processing for histological observation. Hence, the porous osseointegration at the prosthesis-bone interface was evaluated based on histologic and histomorphometric analyses.

**Results:**

At 6 months after surgery, there was no evidence of prosthesis migration, loosening, subsidence, or neurologic or vascular complications. Based on gross histologic analysis, there was excellent porous ingrowth at the prosthesis–bone interface, without significant histopathologic changes. Histomorphometric analysis at the prosthesis-bone interface indicated the mean porous ingrowth of 48.5% ± 10.4% and the total ingrowth range of 36.6 to 59.8%.

**Conclusions:**

As the first comprehensive in vivo investigation into the Prestige LP prosthesis, this project established a successful animal model in the evaluation of cervical disc arthroplasty. Moreover, histomorphometric analysis of porous ingrowth at the prosthesis-bone interface was more favorable for cervical disc arthroplasty with the Prestige LP prosthesis compared to historical reports of appendicular total joint arthroplasty.

**Electronic supplementary material:**

The online version of this article (10.1186/s12891-018-1957-2) contains supplementary material, which is available to authorized users.

## Background

Cervical disc arthroplasty (CDA) is an exciting new technology to treat symptomatic cervical degenerative disc disease in patients who have failed conservative care. This technology not only allows for the maintenance of normal cervical spinal motion, but also has the potential to prevent or reduce the risk of degeneration at adjacent levels, compared with the conventional fusion techniques [1–3]. However, the complications associated with CDA prostheses have been reported as heterotopic ossification, prosthesis migration and dislocation, prosthesis subsidence, spontaneous fusion, immune tissue reaction and fracture in the adjacent vertebral body [4, 5]. Among these, the most important complication is the prosthesis migration and dislocation, because it often requires reoperation. In previous studies, the incidence of prosthesis dislocation after CDA has been reported as 0.5%–3.06% [6–8]. Hence, it is very essential to improve the initial stability and long-term stability of CDA prostheses in order to lower the risk of prosthesis migration and dislocation.

**Fig. 1:**
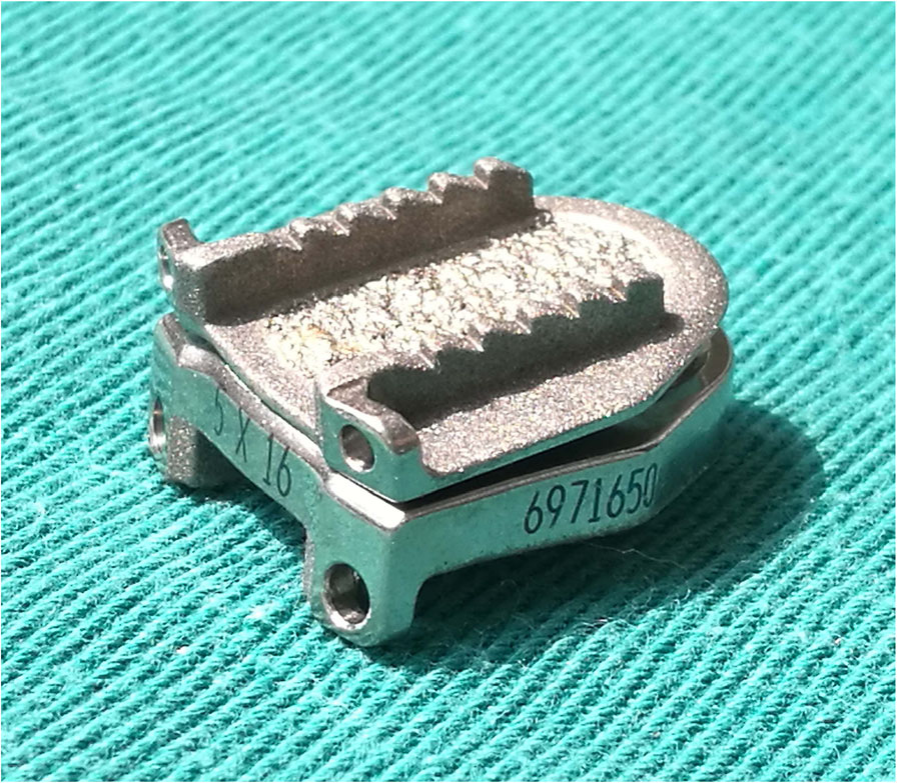
The Prestige LP prosthesis contains two titanium ceramic composite endplates, with two rails on each endplate and a porous titanium plasma spray coating

To date, a variety of commercial and experimental disc prostheses with various design concepts are designed to strive for better initial stability and long-term stability in order to obtain long-term survivorship of CDA prostheses. With regard to the current widely used Prestige LP prosthesis (Medtronic Sofamor Danek, Memphis, TN, USA), with two titanium ceramic composite endplates (Fig. 1), its initial device fixation is mainly attributed to the mechanisms of acute fixation achieved via a series of four rails, two on each prosthesis endplate. Besides, its long-term device fixation is acquired by a porous titanium coating on prosthesis endplate. The primary endplate bearing surface contains two layers of pure titanium, with a pore size of 75–300 μm, which serves to increase porosity and surface area and facilitate trabecular ingrowth [9]. The titanium coatings consist of a special adhesive layer (< 90 μm) and a cover layer (< 180 μm). Owing to these features, a porous titanium plasma spray coating on the endplate contacting surfaces facilitates bone ingrowth and promotes the extent of biological osseointegration at the prosthesis-bone interface, ensuring long-term stability. As it is infeasible to quantitatively evaluate the extent of porous osseointegration at the prosthesis-bone interface for patients underwent cervical disc arthroplasty, an animal model is a great alternative.

Serving as the first to demonstrate successful endplate osseointegration in the caprine cervical model, the present study was undertaken to investigate the initial stability and the biologic porous ingrowth characteristics of the Prestige LP artificial cervical disc prosthesis, with success criteria based on radiographic analysis and quantitative histomorphometry.

## Methods

### Animal research permission

The Institutional Animal Care and Use Committee at the West China Center of Medical Sciences, Sichuan University, Chengdu, Sichuan granted approval for this investigation. Conduct of experimentation on living animals followed the recommendations of the Guide for the Care and Use of Laboratory Animals [10], and under the close supervision of qualified and experienced persons.

### Animal model and surgical preparation

Eight mature male goats (2–3 years old, mean weight 30 Kg) from laboratory animal center of Sichuan University were included in this study, and followed for a period of 6 months after surgery. Each animal was sedated with an intravenous injection of anesthetic medications (diazepam 0.2 mg/kg and ketamine HCL 5 mg/kg), followed by endotracheal intubation and general inhalation anesthesia using 1% to 2% isoflurane with continuous intravenous fluids (range 3–6 mL/lb./h) administered for the duration of surgery. In addition, prophylactic intravenous antibiotics (cefazolin sodium, 1 g) and analgesics (butorphanol 0.1 mg/kg) were administered before and after surgery.

### Surgical technique and postoperative evaluation

**Fig. 2:**
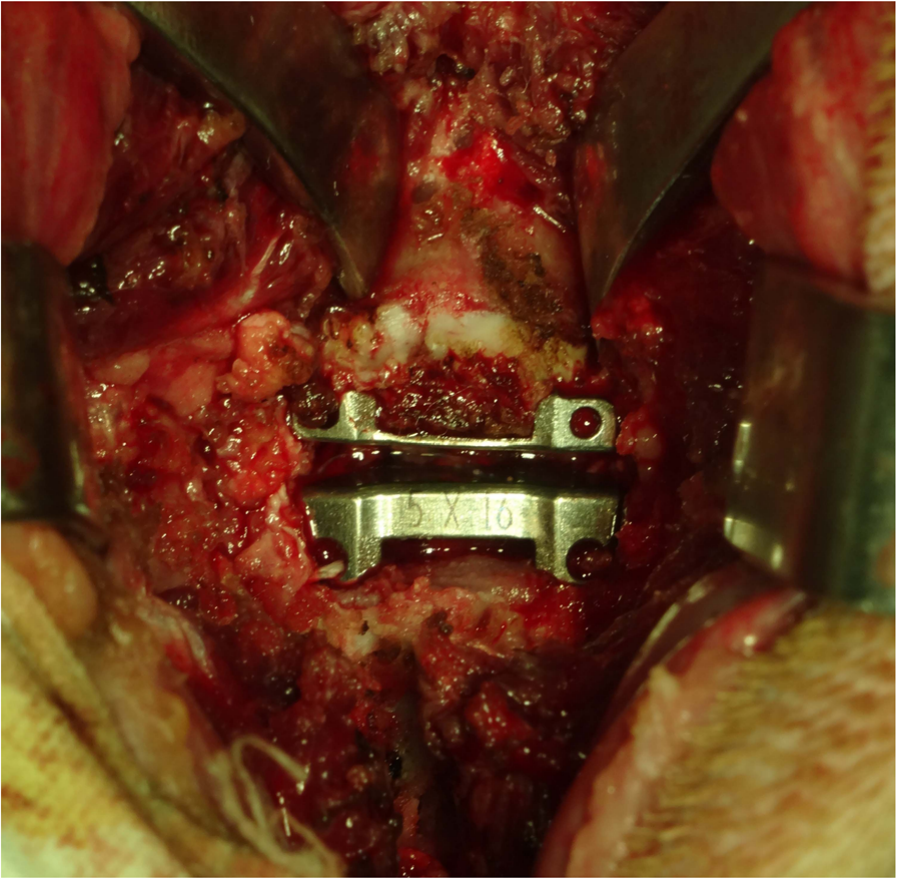
Anterior intraoperative view. The Prestige LP prosthesis is implanted at the surgical level

The anterior Smith-Robinson approach to the cervical spine was adapted to the goat model through a right-sided longitudinal incision with length 6- to 8-cm, and a standard anterior cervical discectomy was performed at the C3–C4 intervertebral level. The endplate surfaces were prepared using curettage and a high-speed burr. According to the manufacturer’s recommended tools and procedures, the Prestige LP prosthesis was then implanted at the operative disc level (Fig. 2). Blood loss, operating times, and intra- and perioperative complications were quantified.

**Fig. 3:**
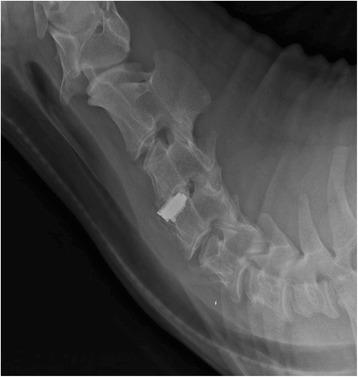
Lateral X-ray film of the caprine cervical spine demonstrates that the disc prosthesis is in place

Observations of ambulatory activities and wound healing were monitored daily, and all animals received analgesics and prophylactic antibiotics for the first 10 days post-operatively. Lateral X-ray films of the cervical spine were taken intra- and post-operatively to confirm the correct position of all disc prostheses (Fig. 3). Euthanasia was performed for each animal using an overdose (150 mg/kg) of concentrated pentobarbital solution (390 mg/mL) at 6 months after surgery. The spinal column then was carefully dissected, immediately placed in double-wrapped plastic specimen bags and frozen at − 20°C for subsequent radiographic and histological examination.

### Histology and histomorphometry

The operative motion segments were examined using microcomputed tomograph (Micro-CT) to histomorphometrically quantify the percentage of trabecular ingrowth at the prosthesis-bone interface. The prosthesis surface was traced manually and expressed as a total endplate area pixel count. The regions of trabecular contact were subsequently traced, quantified in pixels, and expressed as a percentage of the total endplate area (% ingrowth = apparent bone contact area/gross total endplate area). The above quantitative evaluation method has been widely used in several previous studies [11–15].

The vertebral specimens were then performed histological evaluation at the Biotechnology Institute Histology Laboratory of Sichuan University. The specimens were fixed in 10% neutral buffered formalin solution, then dehydrated in a series of graded alcohol which was later substituted by dimethylbenzene, and embedded in paraffin. Thereafter, the paraffin embedded sections were cut into 3–5 μm thick using thin-sectioning microtomy, and then treated with two staining techniques: standard Hematoxylin and Eosin and Masson staining.

### Statistical analysis

Statistical analysis was performed using SPSS version 19.0 software (SPSS Inc., Chicago, Illinois). Histomorphometric data were presented as the percentage of trabecular bone in contact with the Prestige LP prosthesis (titanium endplates) and statistically compared with historical reports of appendicular total joint arthroplasty using an analysis of variance (ANOVA) with Student–Newman–Keuls test. All data were shown as mean ± standard deviation, and significance was indicated at *P* < 0.05.

## Results

All animals survived the procedures and postoperative time period without incidence of vascular, neurologic or infectious complications. The average operating time required was 72.6 ± 17.5 min (range 58–98 min), with an estimated blood loss of less than 50 ml. All animals had a normal recovery by 1 week after surgery, with clinical assessment indicating normal appearance, ambulation, appetite and wound healing. Based on anteroposterior and lateral plain films, there was no evidence of prosthesis migration, loosening, or subsidence at the prosthesis-bone interface.

### Histomorphometry

**Fig. 4:**
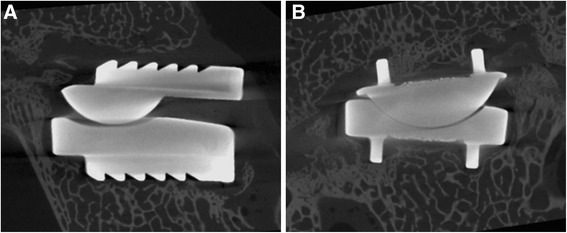
Microcomputed tomograph of the operative segment. Excellent ingrowth is seen at the prosthesis-bone interface at 6 months after surgery in the mid-sagittal plane (**a**) and specified-coronal plane (**b**)

**Fig. 5:**
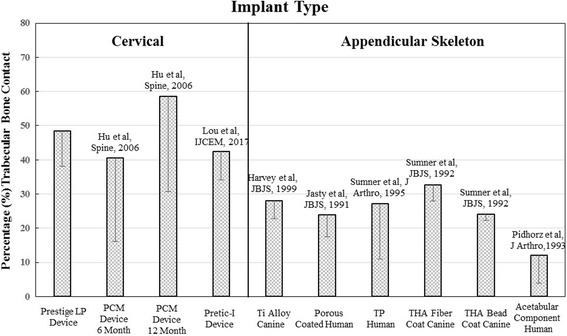
Porous ingrowth. Percentage of ingrowth–bone contact on the endplate surface. The bar graph compares porous ingrowth in cervical disc arthroplasty and appendicular total joint arthroplasty

Micro-CT of the operative motion segments showed excellent osseointegration at the prosthesis–bone interface (Fig. 4a and b). Histomorphometric analysis at the prosthesis-bone interface (apparent bone contact area/gross total endplate area) indicated that the mean porous ingrowth was 48.5% ± 10.4% (total range: 36.6% to 59.8%) at 6 months after surgery, which was higher than that reported for acetabular components, tibial plateaus, and femoral stem components found in the appendicular skeleton (Fig. 5).

### Bone histology

**Fig. 6:**
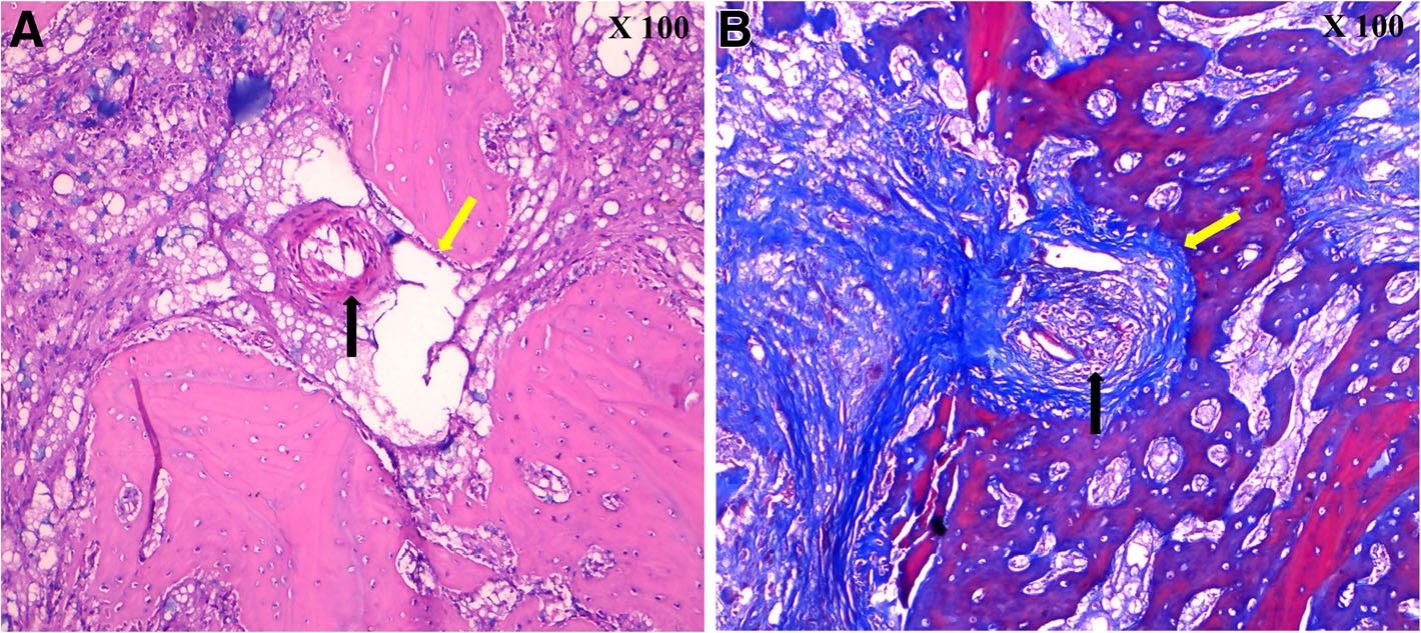
Sections using standard Hematoxylin and Eosin stain (**a**), Sections using Masson stain (**b**). The proliferated osteoblasts (black arrow). The interface between regenerated osseous tissues and mature bone tissues (yellow arrow)

As an overall statement, gross histologic analysis of the Prestige LP prosthesis demonstrated excellent ingrowth at the prosthesis–bone interface, without evidence of particulate wear debris or significant histopathologic changes. All the vertebral specimens were fixed and underwent routine paraffin processing and slide preparation. The paraffin embedded sections were cut, slide mounted, and stained using standard Hematoxylin and Eosin and Masson staining. As a result, there were both plenty of proliferated osteoblasts and regenerated osseous tissues in some regions of the prosthesis-bone interface. Moreover, there was a distinct interface between regenerated osseous tissues and mature bone tissues. (Fig. 6a and b).

## Discussion

To date, a wide variety of commercial and experimental artificial prosthetic discs are now available on the market or are under clinical trial. Despite with different design concepts, all artificial cervical discs are designed to replace the diseased intervertebral discs to perform their functions. The successful outcome of cervical disc replacement, on one hand, rests upon the mechanisms of acute fixation, which are accomplished by endplate modifications involving the use of keels, teeth, rails, and serrations providing initial device fixation [16], on the other hand, depends on the extent of biological osseointegration at the prosthesis-bone interface, ensuring long-term device fixation. Therefore, as a current widely used artificial disc, it is very essential to evaluate the initial stability and the biological porous ingrowth characteristics of the Prestige LP prosthesis.

In the current study, radiographic analysis showed no evidence of prosthesis migration, loosening, or subsidence. Most importantly and most challenging is for device to encourage osseointegration at the prosthesis-bone interface in order to minimize the incidence of clinical device loosening or migration after CDA. Based on the histomorphometry data, the mean porous ingrowth was 48.5% ± 10.4%. This demonstrated excellent porous osseointegration at the prosthesis–bone interface for the Prestige LP prosthesis, similar to that reported in several previous studies [13–15]. Moreover, the mean porous ingrowth was much higher than that reported for porous ingrowth found in appendicular total joint arthroplasty (only 20%–30% ingrowth). Harvey et al. [17] found the values of bone ingrowth to be 9.7 ± 5.38% for a composite stem compared with 28.1 ± 5.31% for a titanium alloy stem in a canine total hip arthroplasty model. Jasty et al. [18] retrieved five porous-coated femoral components from patients underwent revision arthroplasty, and found the ingrowth was 4 to 44% (mean 24%). Sumner et al. [19, 20] investigated the uncemented femoral components at 2 years in a canine total hip arthroplasty model, and found the mean ingrowth to be 32.7 ± 4.7% (range 19.7–47.5%) with fiber metal coatings compared with 24.1 ± 1.8% (range 19.0–31.2%) using bead coatings. Pidhorz et al. [21] retrieved 11 cementless acetabular components at autopsy at an average of 41 months (5 weeks to 75 months) after surgery, with mean ingrowth of 12.1 ± 8.2%. As for the improved extent of porous ingrowth in the Prestige LP prosthesis, one reason may be that the rails provide a better primary press-fit fixation, facing toward the bony endplates to resist pull-out, and the porous titanium plasma spray coating facilitates bone ingrowth and significantly encourages osseointegration at the prosthesis-bone interface in the long run. In addition, we postulate that the more favorable porous ingrowth may be the result of acute ligamentotaxis causing long-term and sustained compression across the prosthesis-bone interface.

There is controversy regarding the most accurate method of measuring the porous ingrowth of cementless prostheses [22–24]. The three most widely used methods are microradiography, stained histology, and backscattered electron imaging-scanning electron microscopy (BEI-SEM). In this study, we adopted microradiography and stained histology to evaluate the porous ingrowth at the prosthesis-bone interface. It turned out that there was evidence of excellent osseointegration at the prosthesis–bone interface. Sumner et al. [25] compared the porous ingrowth of acetabular cups using the three methods, finding that BEI-SEM and histologic sections possessed comparable results, whereas microradiography underestimated the porosity of the porous coating by a mean of 17% and simultaneously overestimated the amount of bony ingrowth by a mean of 0.8%.

There are still some aspects need to be improved in the current study. First, the caprine model used only a 6-month follow-up period and a small sample size. It is expected that longer follow-up evaluation with greater numbers of subjects would be required to obtain a more reliable measure of the biological osseointegration of CDA with the Prestige LP prosthesis. In addition, it is a relatively simplified design for the Prestige LP prosthesis with a porous titanium plasma spray coating. A unique bioactive TiCaPHA (titanium/calcium phosphate/hydroxyapatite) coating is more favorable to encourage osseointegration at the prosthesis-bone interface [13, 14]. Moreover, the prosthesis may possess stronger surface and more favorable porous osseointegration through the procedure for an electrochemically bonded coating than a plasma-sprayed coating [12].

## Conclusions

In summary, as the first comprehensive in vivo investigation into the Prestige LP prosthesis, the present project established a successful animal model for CDA. Moreover, histomorphometric analysis of porous ingrowth at the prosthesis-bone interface was more favorable for CDA with the Prestige LP prosthesis, compared to historical reports of appendicular total joint arthroplasty.

## Additional file

Additional file 1: The original data on the Micro-CT of the operative segments in a caprine cervical disc arthroplasty model. (DOCX 16 kb)

## Abbreviations

BEI-SEM: Backscattered electron imaging-scanning electron microscopy
CDA: Cervical disc arthroplasty
Micro-CT: Microcomputed tomograph
TiCaPHA: Titanium/calcium phosphate/hydroxyapatite
