# Supplementary material for: In-vivo study of osseointegration in Prestige LP cervical disc prosthesis
Source: BMC Musculoskelet Disord. 2018 Feb 7;19:42. doi: 10.1186/s12891-018-1957-2 (PMC5803898; doi:10.1186/s12891-018-1957-2)
Supplement: Additional file 1: — The original data on the Micro-CT of the operative segments in a caprine cervical disc arthroplasty model. (DOCX 16 kb) [file 12891_2018_1957_MOESM1_ESM.docx]

Histomorphometric analysis at the prosthesis-bone interface for 8 mature male goats indicated that the mean porous ingrowth was 48.5% ± 10.4% (total range: 36.6% to 59.8%). Moreover, the porous ingrowth for each goat was as follows: 36.6%, 41.7%, 39.6%, 59.8%, 56.6%, 57.7%, 37.8%, 58.5%. Besides, more data on the Micro-CT for analysis are presented in the following table.

| Sample | BVF (%) | Tb.N (mm^-1^) | Tb.Th (mm) | Tb.Sp (mm) | BMD (g/cm^2^) | |
| --- | --- | --- | --- | --- | --- | --- |
| 1 | 40.48±0.54 | 0.48±0.02 | 0.09±0.01 | 1.11±0.02 | | 0.30±0.01 |
| 2 | 49.07±0.14 | 0.57±0.01 | 0.13±0.01 | 1.20±0.03 | | 0.36±0.01 |
| 3 | 44.83±0.14 | 0.53±0.01 | 0.14±0.01 | 1.18±0.02 | | 0.34±0.01 |
| 4 | 59.27±0.43 | 1.12±0.01 | 0.18±0.03 | 1.44±0.04 | | 0.50±0.01 |
| 5 | 54.49±0.21 | 0.82±0.01 | 0.16±0.04 | 1.31±0.04 | | 0.42±0.01 |
| 6 | 56.19±0.26 | 0.77±0.01 | 0.15±0.04 | 1.35±0.04 | | 0.45±0.03 |
| 7 | 41.97±0.11 | 0.50±0.03 | 0.10±0.01 | 1.13±0.02 | | 0.32±0.01 |
| 8 | 58.39±0.42 | 0.91±0.01 | 0.17±0.04 | 1.38±0.04 | | 0.49±0.02 |

BVF, bone volume fraction; Tb.N, trabecular number; Tb.Th, trabecular thickness; Tb.Sp, trabecular separation; BMD, bone mineral density.
